# Biosorption of Methylene Blue Dye Using Natural Biosorbents Made from Weeds

**DOI:** 10.3390/ma12152486

**Published:** 2019-08-05

**Authors:** Francisco Silva, Lorena Nascimento, Matheus Brito, Kleber da Silva, Waldomiro Paschoal, Roberto Fujiyama

**Affiliations:** 1 Postgraduate Program in Natural Resource Engineering, Federal University of Pará, Belém, PA 66075-110, Brazil; 2 Programa de Pós-Graduação em Física, Universidade Federal do Pará, Belém, PA 66075-110, Brazil; 3 Faculty of Chemistry, Federal University of Pará, Belém, PA 66075-110, Brazil; 4 Department of Natural Sciences; University of the State of Pará, Belém, PA 66050-540, Brazil

**Keywords:** biosorption, weed, methylene blue dye, natural biosorbents, adsorption isotherms, adsorption kinetics

## Abstract

The purpose of this work is to make use of vegetables that, although widely found in nature, there are few applications. The weeds used here, *Cyanthilium cinereum* (L.) *H. Rob* (CCLHR) and *Paspalum maritimum* (PMT) found in the Amazon region of Belém state of Pará-Brazil, contribute to the problem of water contamination by the removal of the methylene blue dye through the biosorption process, taking advantage of other materials for economic viability and processing. The influences of parameters such as, biosorbent dose, contact time, and initial concentration of dye were examined. The characterizations were realized using SEM to verify the morphology of the material and spectroscopy in the FTIR region. As for the adsorption mechanism, the physical adsorption mechanism prevailed. The time required for the system to reach equilibrium for both biosorbents was from 50 min, following a kinetics described by the pseudo-second order model. The adsorption isotherm data for PMT were better adjusted to the Langmuir model and the biosorption capacity ($q_{max}$) value was (56.1798 mg/g). CCLHR was better adjusted to the Freundlich model and its maximum biosorption capacity was 76.3359 mg/g. Thus, these weed species are promising for the biosorption of methylene blue dye in effluents.

## 1. Introduction

The occurrence of weeds in the Amazon Region is considered the most serious biological problem faced by cattle ranchers, as well as their control, one of the highest components of the cost of farms production [1]. It is noteworthy that these plants are undesirable and most of the time they are extracted from nature and discarded or eliminated by chemical processes. Another problem in several countries of the world are the industrial processes that generate significant amounts of effluents containing heavy metals and dyes that affect the quality of water one of the resources the most used by living beings. Water is fundamental to the existence and maintenance of life and for this, it must be present in the environment in appropriate quantity and quality [2].

When colorants are present in aquatic environments, color is generally the first impact to be recognized in an effluent because very small amounts of synthetic dyes in water (<1 ppm) are highly visible [3]. This substance causes serious problems of aesthetic nature in receiving water bodies, even when present in small quantities [4]. Dyes, besides affecting the aesthetic value of water bodies, interfere with the penetration of sunlight into the aquatic environment and thus retard photosynthesis, inhibit the growth of aquatic biotics and interfere with the solubility of gases in bodies of water [5]. In the case of effluents from the textile industries, the dye concentration generally ranges from 10 to 200 mg/L, thus being quite visible [6].

The techniques most commonly used in wastewater treatment are reverse osmosis, ion exchange, adsorption, precipitation [7], membrane filtration [8,9], photocatalysis [10,11,12] and flocculation [13]. Among these methods, adsorption is one of the most effective methods [7,14] and most feasible due to its cost-effective and easy handling [15,16]. Among the adsorbents most applied stand out the zeolites, polymer-based porous materials [17] and, mainly, the activated carbon, most used due to its high surface area, however its use for dye removal is still very expensive, a fact that limits its wide application in the treatment of textile effluents [18]. This has led many researchers to look for more economic and effective adsorbents as potential substitutes for activated carbon [19], resulting in the interest of adsorbents from biomass to be used as sustainable biosorbents. 

It is noteworthy that these plants are undesirable and most of the time they are extracted from nature and discarded or eliminated by chemical processes, which is observed two problematic: one is to give a useful end to vegetal species that in the majority of the times causes disorder to different human activities like agricultural, forestry, animal husbandry, ornamental, nautical, energy production between others [20]. The other is chemical contamination of water which is a worldwide concern, in the case here specified by dyes. Compared with other methods, the removal of dyes from aqueous solutions by the adsorption process proved to be an excellent alternative for effluent treatment, as well as an economical technique [21]. The authors report that the use of biological materials for the removal of dyes from aqueous solutions is commonly referred to as biosorption and has now attracted a great deal of interest in scientific knowledge and in the community as sustainable and ecological materials for the production of alternative sorbents. These materials are called biossorvents [22,23].

Aware of the above problems and the search for solutions for the chemical contamination of water, the objective of this research was to evaluate the biosorption potential of biosorbents produced using weeds as *Cyanthilium cinereum* (L.) *H. Rob* (CCLHR) and *Paspalum maritimum Trin* (PMT), collected in the state of Pará amazon region, aiming at the removal of the methylylene blue dye (MB) from aqueous solutions.

The CCLHR and the PMT were characterized by Scanning Electron Microscopy to investigate their morphologies and by Fourier transform infrared spectroscopy (FTIR) to detect the presence of functional groups present in the material that corroborate for their use in the removal of methylene blue (MB) of aqueous solution. PMT is a native species of tropical America, occurring in Central America and the Caribbean, northern Brazil and the coastal zone, from Northeast to South. In Brazil the highest concentrations occur from Pará to Bahia [24]. The CCLHR also known as Vernonia cinerea belongs to the Asteraceae family. The species is native to tropical Africa, tropical Asia, India, Indochina, tropical South America, West India and the US state of Florida [25].

Recent studies indicate that approximately 12% of the synthetic dyes are lost during manufacturing and processing operations and that about 20% end up entering the environment through effluents from industrial wastewater treatment plants [26]. Dyes have a complex chemical structure that is stable to light, heat, oxidizing agents and are also resistant to aerobic digestion [27,28]. Methylene blue is a cationic dye widely used in the textile industry for the dyeing of cotton and wool fabrics. When untreated, uncontrolled discharge into rivers and lakes affect not only the transparency of the waters, but also limits the passage of solar radiation, reducing the natural photosynthetic activity and causing changes in the aquatic biota and causing acute and chronic toxicity of these ecosystems [29,30].

As has already been synthetically mentioned, an appropriate alternative method, which has proved to be quite effective for removal of dyes from aquatic environments is biosorption, a subcategory of adsorption, which uses as biological raw material where the lingnocellulosics are included. In this class of materials, agricultural byproducts have been shown to be efficient because, in addition to being abundant, they are inexpensive and have a relatively low impact on the environment. In comparison to other effluent treatment methods, biosorption substantially reduces the costs associated with financial investment in the process as a whole [31,32].

Several works that deal with the removal of methylene blue are presented in the literature using biosorbents obtained from lignocellulosic materials in the in natura form such as coconut fiber [33], banana peel [34], mint tailings [35], pineapple peel [36], cashew nutshell [37] pine leaves [38], tea residues [39], corn straw, pupunha palm [40], trunk of the papaya tree [41]. In this paper, the biosorbents presented for the removal of methylene blue dye (MB) were produced from weeds that can be defined as any plants that grow spontaneously in a place of human activity and cause damage to this activity, be it agricultural, forestry, livestock, ornamental, nautical, energy production etc. [42].

This work has objective produce biosorbents through weeds (PMT and CCLHR) and realize biosorptions test to verify removal efficiency MB from aqueous solutions. In the biosorption assays, the influence of parameters such as dosage of biosorbents between (0.05–0.5 g), initial concentration of dye in the range of (10.0 and 50.0 mg/L), and contact time (10 and 80 min) were evaluated. The experimental data of biosorption isotherms were evaluated by the Freundlich and Langmuir models and biosorption kinetics by the pseudo-first-order and pseudo-second-order models. The maximum biosorption capacity ($q_{max}$) values were 56.1798 mg/g for the PMT, whose experimental biosorption isotherm data were better adjusted with the Langmuir model and 76.3359 mg/g for the CCLHR, whose experimental data of biosorption isotherms were better fitted to the Freundlich model. Finally, the research result shows the biosorbent’s potential for removal MB from waste water. It is expected that this research will contribute as an alternative in the problematic of the water chemical contamination, using a simple reproduction process and a wide availability of raw material for biosorbents production.

## 2. Materials and Methods 

### 2.1. Preparation of Biosorbent

The used biosorbent were produced from weeds (*Cyanthilium cinereum* (L.) *H. Rob e Paspalum maritimum Trin*). The weeds found in the Amazon region, Belém-PA, Brazil. They were collected manually within the Universidade Federal do Pará (UFPA). After harvesting, the stems were extracted and cut into lengths of approximately 5 mm. The trimmed stems were washed in distilled water and introduced in an oven at 400 °C for 30 days, to reduce humidity and to avoid attack of microorganisms. After 30 days, the dried samples were again crushed and washed with distilled water until we did not observe more of the coloration in the solution. The wet samples were placed in an oven at 800 °C for 24 h. The prepared biosorbents from weeds were stored in airtight plastic containers in order to avoid humidity, and these were utilized in the biosorption assays.

### 2.2. Solutions and Reagents

In the present study of biosorption, the used adsorbate was the methylene blue dye (MB), classified as basic or cationic. A stock solution of 100 mg/L was prepared separately, which was diluted to between 10 and 50 mg/L. The molecular structure and chemical formula of the Cationic dye of methylene blue are shown in Table 1.

### 2.3. Used Equipments at the Characterization

In the Fourier Transform Infrared Spectroscopy (FT-IR) of the biosorbents was used Perkin Elmer Spectrum Two, in order to verify functional groups present in the samples. The biosorbents morphological was investigated by using Tescan scanning electron microscope (SEM) (Vega3 SB).

### 2.4. Biosorption Experimental Procedure

The biosorption is biomass ability to adsorb surface pollutants by carboxylic and phenolic functional groups, in which neutral pH make deprotonated and negative charge removes cations from solution by means of process as complexation, ionic exchange and adsorption [43].

The biosorption studies for the assessment of weeds (*Cyanthilium cinereum* (L.) *H. Rob* and *Paspalum maritimum Trin*) for the removal of MB dye from aqueous solutions was conducted by means of the batch biosorption procedure using 50 mL–pH 7 of solution, submitted to constant agitation speed of 150 rpm by magnetic stirrer (QUIMIS–Q221 MAG model), without temperature control. We analyzed the influence of parameters such as biosorbent dosage between 0.05–0.5 g, initial dye concentration in the range of 10.0 and 50.0 mg/L and contact time between 10–80 min. During each procedure at predetermined time intervals, solution samples were taken for residual analysis of MB concentrations using spectrophotometer. Equations (1) and (2) were used to calculate the percentage of removal and the biosorption capacity, respectively:

(1) $$R\%=\frac{\left(c_{0}-c\right)}{c_{0}}\;\times 100\%$$

(2) $$q\left(t\right)=\frac{\left(c_{0}-c\right)}{m}\;\times V$$

where:

R% = Percentage of removal
$c_{0}\;$ = Initial concentration (mg/L)
c = Concentration at time t
V = Volume (L)
$q\left(t\right)$ = Biosorption capacity at time t
*m* = Biosorbent mass.

## 3. Results

### 3.1. Characterization of PMT and CCLHR Biosorbents

**Figure 1:**
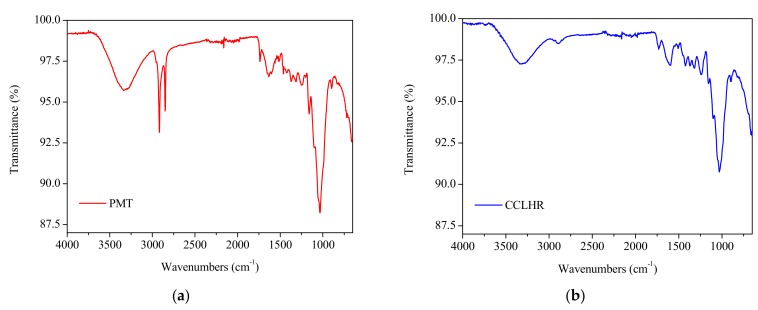
Infrared spectrum of *Paspalum maritimum* (PMT) (**a**) and CCLHR (**b**) samples, in the range of 400–4000 cm^−1^.

Figure 1 shows the FTIR results for the identification of the present functional groups in the PMT and CCLHR species. In the range of 3000–3720 cm^−1^, a broad and low intensity band was observed, peaks at 3330 cm^−1^ (Figure 1a) and 3320 cm^−1^ (Figure 1b), characterizing the presence of O–H (alcohols and phenols) [44]. Peaks were also observed at 1630 cm^−1^ (Figure 1a) and 1613 cm^−1^ (Figure 1b), which indicated the C = O stretching in organic groups of carboxyl and bending vibration of the functional group –OH [45]. The intense band at 1035 cm^−1^ (Figure 1a) and 1033 cm^−1^ (Figure 1b) peaks confirm the functional groups O–C–O of the cellulose and lignin structure.

The biosorption of MB on the adsorbent may be due to the electrostatic attraction between these groups and the cationic dye molecule. At pH above 4, the carboxylic groups are deprotonated and negatively charged carboxylate ligands (–COO–) bind to the positively charged MB molecules. This confirms that the biosorption of MB by adsorbent was an ion exchange mechanism between the negatively charged groups present in adsorbent and the cationic dye molecule [46].

**Figure 2:**
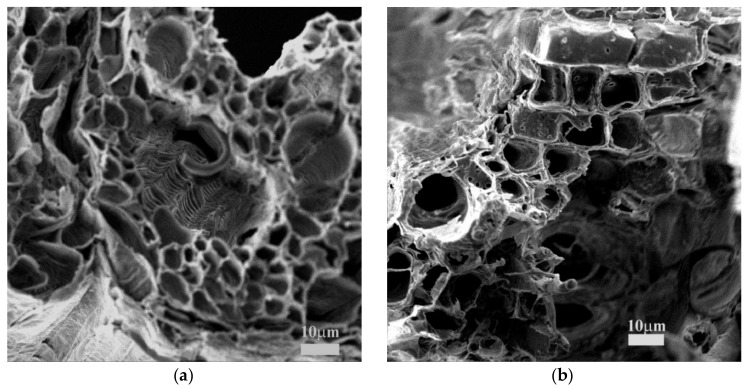
Scanning electron microscope (SEM) image of (**a**) PMT with magnification of 2190× and (**b**) CCLHR with magnification of 1720×.

The Scanning Electron Microscopy images of PTM and CCLHR are shown in Figure 2 with magnification of 2190× and 1720×, respectively The SEM analyzes showed a large amount of pores implying a wide surface area, which facilitates the MB biosorption process [40]. This indicates a necessary requirement of these lignocellulosic materials as potential biosorbents. In general, the PMT and CCLHR presented different morphologies along their surface demonstrating different pores sizes and heterogeneous surfaces.

The characterization results by SEM and FTIR show the predominance the physical adsorption mechanism due to the porosity of the material and the electrostatic interaction between the biosorbent and the methylene blue.

### 3.2. The Effect of Dosage

**Figure 3:**
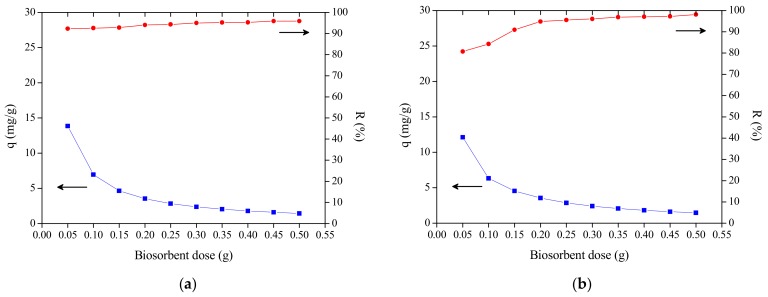
Biosorbent dose vs. biosorption capacity vs. percentage of removal: (**a**) PMT and (**b**) CCLHR.

The biosorption capacity represent the biosorbate mass can be retained by the biosorbent mass while percentage of removal is related to speed which the biosorbate flows from solution to biosorbent surface. The MB biosorption by the CCLHR and PMT plant species was examined by dosage variation of 0.05 to 0.5 g in the concentration 15 mg/L, solution volume of 50 mL and agitation speed 150 rpm. For both species, we observed that the increase in the mass of produced biosorbents led to an increase at the percentage of removal, with CCLHR from 80.96% to 98.15% and PMT from 92.33% to 95.88%. However, increasing the dosage from 0.05 to 0.5 g caused a decrease in biosorption capacity, where for CCLHR form 12.11 to 1.47 mg/g, whereas for PMT from 13.85 for 1.44 mg/g. The increase in the percentage of removal with increasing dosage is because the larger amount of mass provided a larger number of active sites available for the biosorption, which causes the increase of the percentage of removal as already reported in the literatures [47,48]. The decrease of the biosorption capacity with the increase of the biosorbent dosage can be explained by the unsaturation of a certain number of active sites, since the volume and concentration remained fixed to a higher mass value, in which should be distributed the same amount of dye. Also, there is the particle aggregation due to the increase of the biosorbent dosage, which causes a decrease in the surface area and the increase of the diffusion path to be travelled by the adsorbate inside the biosorbent [48,49,50,51,52,53,54]. Figure 3 shows the curves of biosorbent dose vs. biosorption capacity and biosorbent dose vs. percentage of removal of PTM and CCLHR.

### 3.3. Effect of Initial Dye Concentration

**Figure 4:**
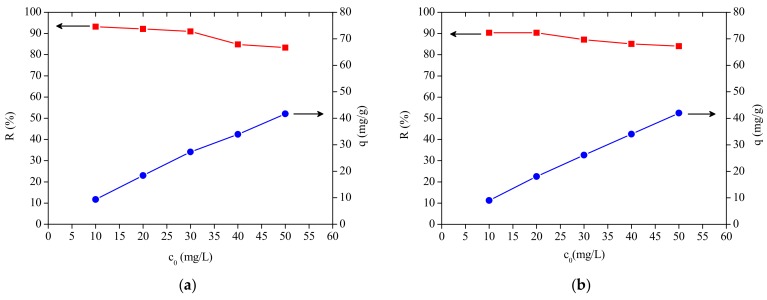
Initial concentration vs. percentage of removal vs. biosorption capacity: (**a**) PMT and (**b**) CCLHR.

The initial dye concentration in the range of 10–50 mg/L was studied for the assessment of MB biosorption using a biosorbent dosage of 0.05 g. The biosorption capacity increased with increasing concentration from 9.03 to 41.99 mg/g (MB) for CCLHR biosorbent and from 9.31 to 41.67 mg/g (MB) for PMT biosorbent. The percentage of removal decreased from 90.36% to 83.98% for CCLHR biosorbent and from 93.18% to 83.35% for PTM biosorbent. At lower initial concentrations of MB there are relatively few dye molecules and a large number of available adsorption sites, which are present in the biosorbent masses, thus, it leads to a better interaction of the adsorbate with the biosorption sites, hence resultant to the higher percentage of MB removal. With the increase in the initial concentrations of MB occurs the gradual decrease in the percentage of removal due to the saturation of active biosorbents sites, since with the elevation of concentration the number of the dye molecules increases significantly [55]. The increase of initial dye concentration from 10 mg/L to 50 mg/L provided an increase in the biosorption capacity, since higher concentrations contribute to a decrease in the adsorbate mass transfer resistance of solution to the adsorbent surface, thus filling possible active sites still unoccupied in low concentrations [56,57] (Figure 4).

### 3.4. Adsorption Isotherms

Isotherms are diagrams showing the variation of equilibrium concentration of adsorbent with the liquid phase concentration at a temperature. These models are used to illustrate the biosorbent interaction with the biosorbate and provide the relationship between the biosorption capacity and the liquid phase concentration of biosorbate under equilibrium condition at constant temperature [58].

#### 3.4.1. Langmuir Isotherm

The Langmuir model is used in the investigation of dye biosorption from liquid solution [41]. The model based on the assumption that exists a defined number of active sites, the biosorption process occurs on a homogeneous surface through monolayer formation without any interaction with the biosorbed molecules and that all the sites has equivalent energy [56,59,60] The Langmuir isotherm [61] is represented by Equation (3). 

(3) $$q_{e}=\frac{q_{max}k_{L}c_{e}}{1+k_{L}c_{e}}$$

Where:

$q_{e}$ = amount of solute adsorbed per gram of adsorbent at equilibrium (mg/g);
$q_{max}$: maximum biosorption capacity (mg/g);
$k_{L}$ interaction constant of adsorbate/adsorbent (L/mg);
$c_{e}$: equilibrium concentration of adsorbate (mg/L).

From Equation (3), we can obtain the linearized form:

(4) $$\frac{c_{e}}{q_{e}}=\frac{1}{q_{max}}c_{e}+\frac{1}{k_{L}q_{max}}$$

And so, plot a chart $c_{e}/q_{e}$ in function of $c_{e}$ which allows you to calculate the which allows you to calculate the $q_{max}$ e $k_{L}$ being that $\;\;1/q_{max}$ is the angular coefficient of the line and the $1/k_{L}q_{max}$ is the intercession with the ordinate axis.

In Langmuir’s model a widely used indicator in terms of analysis is called the separation factor $R_{L}$ which is calculated on the basis of $c_{0}$ e $k_{L}$ according to Equation (5).

(5) $$R_{L}=\frac{1}{1+k_{L}c_{0}}$$

$c_{0}=\;$ initial concentration (mg/L).

The value of the constant $q_{max}$ is related to the adsorbed species concentrations on the surface. When the biosorption capacity reaches this value it means that all available sites (sites that the adsorbate binds to the adsorbent) have been filled. The constant $k_{L}$ is related to the free energy of adsorption, which corresponds to the affinity between the surface of the adsorbent and the adsorbate [58]. $R_{L}$ indicates the mode of adsorbate biosorbent interaction and allows to classify adsorption isotherms in unfavorable ($R_{L}\;$> 1), linear ($R_{L}$ = 1), favorable (0 < $R_{L}$ < 1), or irreversible ($R_{L}$ = 0) [62].

#### 3.4.2. Freundlich Isotherm

According to Freundlich model, the multilayer adsorption occurs on heterogeneous adsorbent surfaces and the higher energy sites on the surface are first occupied and the binding force decreases with the increase in the degree of occupation of the active sites, which reduces the adsorption with time [56,59].

The Freundlich model is represented by [63]:

(6) $$q_{e}=k_{F}c_{e}{}^{\frac{1}{n}}$$

According to Cai L. et al. [44], the linearized form of Equation (6) is described by:

(7) $$\log q_{e}=\log k_{F}+\frac{1}{n}\log c_{e}$$

$k_{F}$ and n are the Freundlich constants related to biosorption capacity and biosorption intensity, respectively.

**Figure 5:**
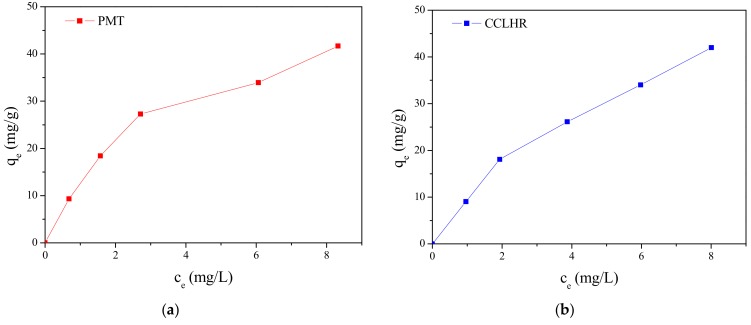
Adsorption isotherm of MB: (**a**) PMT, (**b**) CCLHR.

**Figure 6:**
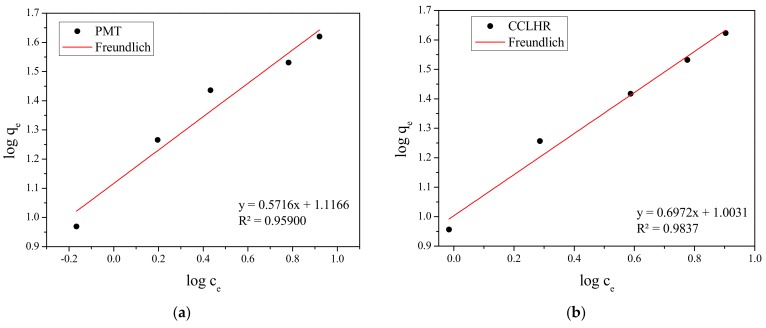
Freundlich isothermal adsorption equation fitting of methylene blue: (**a**) PMT, (**b**) CCLHR.

**Figure 7:**
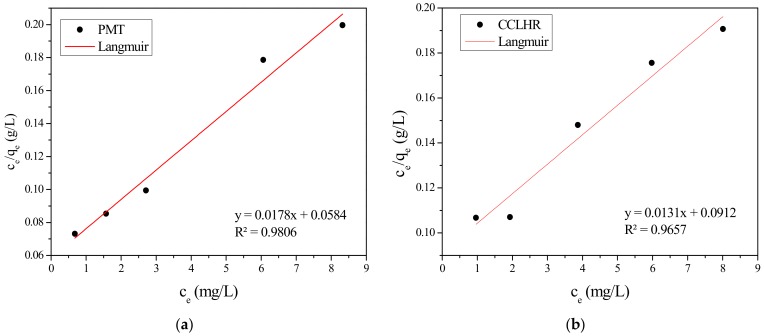
Langmuir isothermal adsorption equation fitting of methylene blue: (**a**) PMT, (**b**) CCLHR.

**Figure 8:**
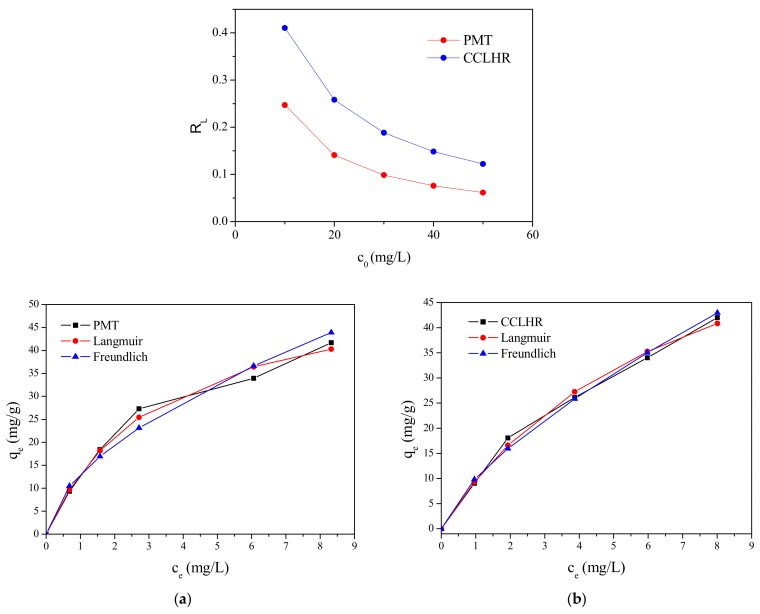
Adsorption isotherms with the Langmuir and Freundlich models: (**a**) PMT, (**b**) CCLHR.

The adsorption isotherms obtained experimentally for PMT and CCLHR (Figure 5) were adjusted to the Langmuir and Freundlich models, for initial concentrations in the range between 10 and 50 mg/L. The values of the constants were calculated by the linearized forms of the respective models. Figure 6 and Figure 7 show Freundlich and Langmuir models for both biosorbents (PMT and CCLHR), respectively. The results for the parameters obtained in both biosorbents are shown in Table 2. Analyzing the values of the correlation coefficient in Table 2 and Figure 8, we observed that the experimental data of the adsorption for the PMT is better adjusted to the Langmuir model, while the CCLHR to the Freundlich model. It is justified by the values of the respective linear correlation coefficients (R^2^ closer to unity) in each case.

In relation to the constants $k_{L}$, $q_{max}$ and 1/n (highest value of n) for the PMT and CCLHR Table 2 we noticed that the highest value of $k_{L}$ and lower value of 1/n were found for the PMT, although the higher value of $q_{max}$ was for CCLHR. Both models indicated higher MB affinity for PMT surface. The higher value of $q_{max}$ for the CCLHR was possibly due to the fact of the greater diffusion in the pores and intraporos of the CCLHR than PMT. Therefore, the CCLHR presented better efficiency in relation to PMT at the conditions investigated in this work.

Various biosorbents have been applied in removal MB from the aqueous solution, as reported in the previous literature, for comparison purposes. We can compare the results with other authors in term the maximum capacity adsorbed in the conditions optimized by each authors: Carica papaya wood $(q_{max}=32.25\;\mathrm{mg}\cdot g^{-1}$) [41], Cornbread $\left(q_{max}=106.383\;\mathrm{mg}\cdot g^{-1}\right)$ and pupunha palm $\left(q_{max}=78.989\;\mathrm{mg}\cdot g^{-1}\right)$ [40], Potato shell $\left(q_{max}=48.7\;\mathrm{mg}\cdot g^{-1}\right)$ [64], Scenedesmus $\left(q_{max}=61.69\;\mathrm{mg}\cdot g^{-1}\right)$ [65]. Through the comparative study with the Table 2, we can conclude that PMT and CCLHR are between the most efficient adsorbents prepared for industrial wastewater treatment.

**Figure 9:**
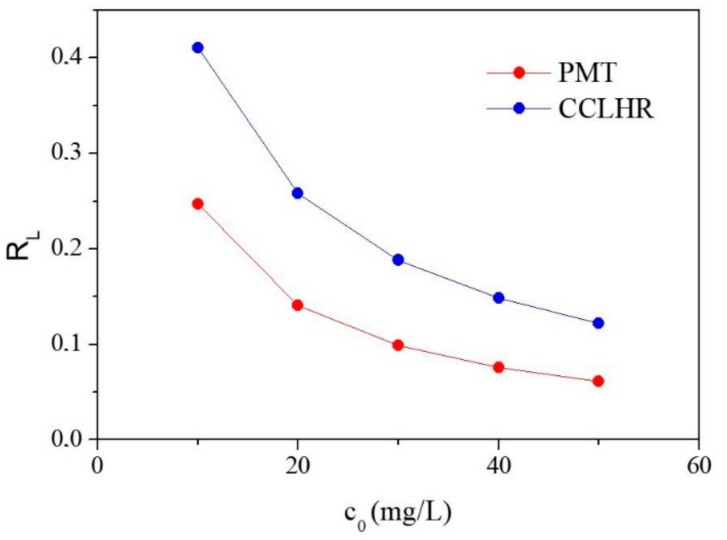
Curve of R_L_ by the initial concentration for PMT and CCLHR.

The values of $R_{L}$ between 0 and 1 Langmuir model, whose variation with the initial concentration is shown in Figure 9, for both biosorbents and of n between 1 and 10 Freundlich model are in Table 2, confirm that the biosorption was favorable for both the biosorbents (PMT and CCLHR), ie, the adsorbate prefers the solid phase than liquid [58].

### 3.5. Adsorption Kinetics

**Figure 10:**
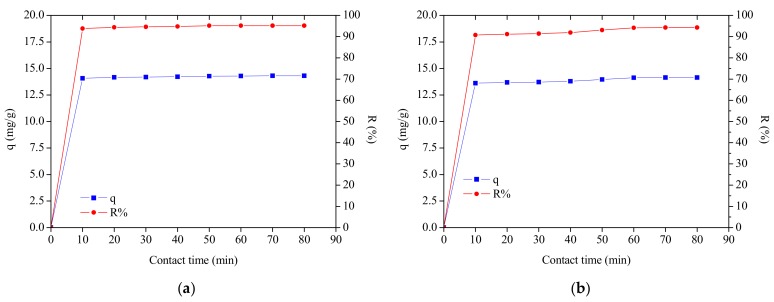
Contact time vs. percentage of removal vs. biosorption capacity: (**a**) PMT and (**b**) CCLHR.

The biosorption with the interaction time between the biosorbents and MB was evaluated in the range of 10 to 80 min, a concentration of 15 ppm and a mass of 0.05 g was used. According to Figure 10, we observed that in the first 10 min occurred a rapid increase in the percentage of removal and percentage of biosorption by both biosorbents. After this period, they became slower and remained practically constant from 50 min. The results are due to the fact that in the initial phase of the biosorption, the dyes particles to be biosorbed were almost entirely present in the solution with high probability of accessing the biosorbents surface and the active sites are unoccupied at the beginning of the process. With the increased of the time occurred a decrease in the concentration due to the migration of MB to unoccupied sites, which hindered the biosorption process by increasing the competition of residual dye particles for the remaining available sites. Results in terms of this behavior have already been reported in the literature [59,66,67,68].

In the literature, there is an expressive amount of linear kinetic models that are used to evaluate the controlling mechanism of the biosorption process such as the chemical reaction, diffusion and mass transfer [69]. Frequently, the most used models are pseudo-first-order and pseudo-second-order, which were also used in this work. The equations and their linearized forms are shown in Table 3.

**Figure 11:**
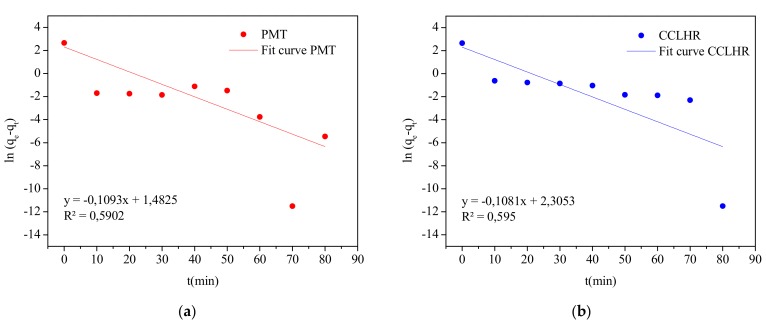
Plots of pseudo-first-order kinetic model for the biosorption: (**a**) PMT and (**b**) CCLHR.

**Figure 12:**
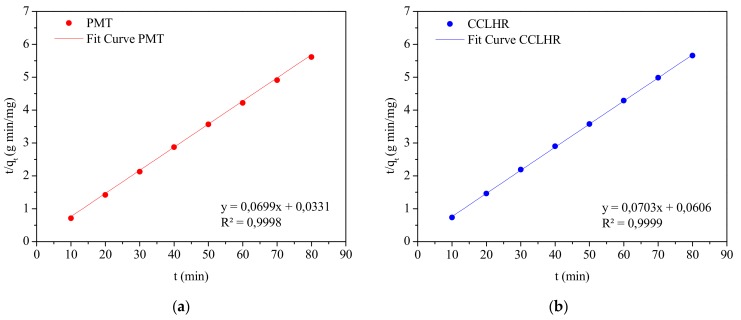
Plots of pseudo-second-order kinetic model for the biosorption: (**a**) PMT and (**b**) CCLHR.

In order to confirm the experimental data, we utilized the pseudo-first-order and pseudo-second-order models, where kinetic biosorption parameters of MB for initial concentration of 15 ppm are shown in Table 4. In view of these results, it was observed that for both biosorbents the model that best represented the experimental data was the pseudo second order, since R² is closer to unity. Figure 11 and Figure 12 show the behavior of the linearized form of said models through which the parameters of Table 4 were calculated.

## 4. Conclusions

The characterizations by FTIR and SEM showed, respectively, functional groups (hydroxyl, carbonyl and carboxyl) and high porosity surface, these factors confirm the produced biosorbents by PMT and CCLHR weeds present appropriate physical-chemical properties to adsorption process. The adsorption kinetics showed high remove percent and experimental data were most be adjusted by pseudo-second-order model. The maximum biosorption capacity (*q_max_ =* 56.1798 mg·g^−1^ and *q_max_ =* 76.3359 mg·g^−1^ for PMT and CCLHR, respectively) showed equivalent biosorption value to literature used by MB removal from industries wastewater. The experimental data of the adsorption for the PMT is better adjusted to the Langmuir model, while the CCLHR to the Freundlich model. The range *R_L_* and *n* results indicated favorable biosorption. Overall, the raw material showed potential for applications with low cost biosorbent, can be a viable alternative and had and with ecological appeal to remove Methylene Blue dyes by several industries segments.

## 5. Patents

In this work, the used plant raw materials are filed with the National Institute of Industrial Property (INPI)-Brazil, with the code BR 10 2019 008806-0. Additionally, eight weed species divided among the Cyperaceae, Poaceae, Amaranthaceae, Asteraceae families have already been tested and showed biosorption potential.

## Figures and Tables

**Table 1:** Dye characteristics.

Cationic Dye	Molecular Structure	Chemical Formula
Methylene blue	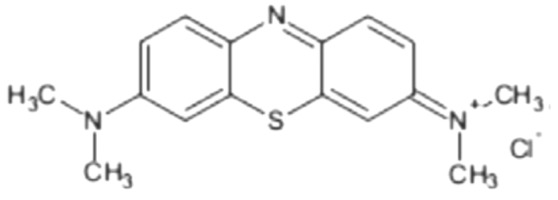	C_16_H_18_N_3_SCl

**Table 2:** Adsorption isotherm parameters for methylylene blue (MB).

Sample	Freundlich			Langmuir			
	*n*	*k_F_*	*R²*	*q_max_* (mg/g)	*k_L_*	*R²*	*R_L_*
PMT	1.7495	13.0798	0.9590	56.1798	0.3048	0.9806	0.2470–0.0616
CCLHR	1.4343	10.0716	0.9837	76.3359	0.1436	0.9657	0.4104–0.1222

**Table 3:** Kinetic equations of biosorption.

Model	Equation	Linearized Formula
Pseudo-first-order	$dq_{t}/dt=k_{1}\left(q_{e}-q_{t}\right)$	$\ln\left(q_{e}-q_{t}\right)=\ln q_{e}-k_{1}t$
Pseudo-second-order	$dq_{t}/dt=k_{2}\left(q_{e}-q_{t}\right)²$	$\frac{t}{q_{t}}=\;\frac{1}{k_{2}q_{e}^{2}}+\frac{t}{q_{e}}$

**Table 4:** Comparison of the pseudo-first-order and pseudo-second-order models for the biosorption of MB on PMT and CCLHR.

Kinetic Model	Parameter	PMT	CCLHR
Pseudo-first-order	k_1_ (mg·min/g)	0.1093	0.1081
	q_e_ (mg/g)	14.2554	14.1435
	R^2^	0.5902	0.5950
Pseudo-second-order	k_2_ (mg·min/g)	0.1476	0.0815
	q_e_ (mg/g)	14.3062	14.2248
	R^2^	0.9998	0.9999
